# Improving the Robustness of Object Detection Through a Multi-Camera–Based Fusion Algorithm Using Fuzzy Logic

**DOI:** 10.3389/frai.2021.638951

**Published:** 2021-05-26

**Authors:** Md Nazmuzzaman Khan, Mohammad Al Hasan, Sohel Anwar

**Affiliations:** ^1^ Department of Mechanical and Energy Engineering, Indiana University Purdue University Indianapolis, Indianapolis, IN, United States; ^2^ Department of Computer and Information Science, Indiana University Purdue University Indianapolis, Indianapolis, IN, United States

**Keywords:** object detection, multi-camera, confidence score, fuzzy logic, sensor fusion

## Abstract

A single camera creates a bounding box (BB) for the detected object with certain accuracy through a convolutional neural network (CNN). However, a single RGB camera may not be able to capture the actual object within the BB even if the CNN detector accuracy is high for the object. In this research, we present a solution to this limitation through the usage of multiple cameras, projective transformation, and a fuzzy logic–based fusion. The proposed algorithm generates a “confidence score” for each frame to check the trustworthiness of the BB generated by the CNN detector. As a first step toward this solution, we created a two-camera setup to detect objects. Agricultural weed is used as objects to be detected. A CNN detector generates BB for each camera when weed is present. Then a projective transformation is used to project one camera’s image plane to another camera’s image plane. The intersect over union (IOU) overlap of the BB is computed when objects are detected correctly. Four different scenarios are generated based on how far the object is from the multi-camera setup, and IOU overlap is calculated for each scenario (ground truth). When objects are detected correctly and bounding boxes are at correct distance, the IOU overlap value should be close to the ground truth IOU overlap value. On the other hand, the IOU overlap value should differ if BBs are at incorrect positions. Mamdani fuzzy rules are generated using this reasoning, and three different confidence scores (“high,” “ok,” and “low”) are given to each frame based on accuracy and position of BBs. The proposed algorithm was then tested under different conditions to check its validity. The confidence score of the proposed fuzzy system for three different scenarios supports the hypothesis that the multi-camera–based fusion algorithm improved the overall robustness of the detection system.

## 1 Introduction

Real-time weed detection is an emerging field where agricultural robots apply deep neural networks for real-time weed detection, crop management, and path planning extensively (Vougioukas, 2019; Wang et al., 2019). With the emergence of agricultural robotics, accurate identification of weeds and crops with deeper understanding of overall object detection technology becomes more important. In general, object detection systems detect a target object using classical computer vision and deep learning techniques, determine the category of the detected object, and create a bounding box marking the position of the object (Russakovsky et al., 2015; Zhiqiang and Jun, 2017). But real-time object detection is a complex challenge due to the effect of background, noise, occlusion, resolution, and scale affecting the performance of the system (Zhiqiang and Jun, 2017). In 2013, R-CNN (regions with CNN features) (Girshick et al., 2014) showed much improvement in object detection compared to conventional computer vision techniques and started the trend on CNN-based object detection.

Many state-of-the-art convolutional neural network (CNN) detector models like SPP-net (He et al., 2015), Fast R-CNN (Girshick et al., 2014), YOLO (Redmon et al., 2016), and RetinaNet (Lin et al., 2017) improved detection accuracy on standard image datasets using new CNN architectures. But in most of the cases, CNN models are trained with images without noise or degradation. During training, image augmentation is used to introduce noise to increase the robustness of the model, but sometimes, they may fail to capture the real scenario. It is not possible to include all probable types of noise during the training of a CNN. In the real scenario, noise can affect the quality of image due to sensor quality, lighting, vibration, exposure time, *etc*. It is already shown that introducing carefully selected noise can produce wrong results even though they have no effect on visual recognition (Moosavi-Dezfooli et al., 2016). Prasun et al. (Roy et al., 2018) showed how different image degradations can affect the performance of CNN models. They were unable to come up with a solution which can produce a robust CNN architecture against image degradation when a large number of classes are present, such as ImageNet. Moreover, in recent times, it is observed that the accuracy of CNNs reduces significantly when only tested on negative images, which shows an inherent bias toward positive training dataset (Hosseini et al., 2017). In this research, we want to address this inherent limitation of CNN regarding uncertainty toward correct object detection and bounding box (BB) creation with a multi-camera setup and fuzzy logic.

Fuzzy logic has high potential in understanding complex systems where analytical solution may not exist or the system is not understood properly but can be observed (Ross et al., 2004). According to Ross, fuzzy systems are useful in two scenarios: “1) in situations involving highly complex systems whose behaviors are not well understood and 2) in situations where an approximate, but fast, solution is warranted” (Ross et al., 2004). In agricultural robotics, fuzzy neural network–based sliding mode control is used to build an apple picking robot (Chen et al., 2019). Romeo et al. used fuzzy clustering with dynamic threshold for greenness identification (Romeo et al., 2013). Meyer et al. used fuzzy clustering for classifying plant and soil from color images (Meyer et al., 2004). A fuzzy classifier is used to detect weeds in real time in sugarcane fields (Sujaritha et al., 2017). A fuzzy expert system is used in soil management (López et al., 2008), to predict cotton yield (Papageorgiou et al., 2011), and in crop disease management with a text-to-talk user interface (Kolhe et al., 2011). In general, fuzzy logic is used as a classifier for crop or weed detection and as an expert system for crop, weed, and soil management. But no thorough research studies are found which used fuzzy logic to improve weed detection accuracy using a multi-camera setup.

The goal of this research was to implement a multi-sensor–based fuzzy fusion algorithm to improve the robustness of any CNN-based object detection system. In this article, Section 2 presents the research steps. Section 3 shows the experimental setup of the multi-camera system. It also shows how the homography matrix is calculated for this setup. Section 4 describes the CNN-based object detection method with classification results. Section 5 describes the fuzzy rules and membership functions. Section 6 shows the results of the fuzzy fusion system.

## 2 Methodology

Weed can be detected with a CNN-based detector with very high accuracy and in real time. If we use multiple cameras, then the robustness of the overall system increases. When we use a detector to create a BB, it gives us the accuracy percentage of objects inside the BB and the position of the BB. In this article, we explore the possibility of improving the overall system robustness by measuring the “confidence” of the BB position of multiple cameras. The detector will give us the position of BB on each image plane. But how do we know for sure that the position of the BB is correct? When we are detecting an object using multiple cameras, the position of the BB on the camera image plane may appear in different places based on the detection accuracy and the position of the cameras. But in 3D space, the object is at the exact same position for both cameras. We will manipulate this information to calculate the confidence score of the BB position using projective transformation, IoU overlap value, and fuzzy logic–based fusion. We will complete the following steps to calculate the confidence score:

• Create a two-camera setup and calculate the homography between the two cameras.
• Place weed (object) at different specific distances from the camera setup.
• Use a CNN detector to create BB of detected weed on both cameras. Use best possible detection BB so that the weed is perfectly detected and inside the BB.
• Use the homography matrix to project one camera image plane over another, and then calculate the IoU overlap values of the BBs after projection for different weed positions.
• The logic is as follows: if perfect BB is created at a certain distance of weed position, then it will have a specific IOU overlap value. We will have “high” confidence for this scenario. If the position or IOU value deviates from the perfect condition, then we will have “less than high” confidence on the detection. Use fuzzy “IF...THEN” rules to capture these conditions.
• Use defuzzification to get the crisp “confidence” value for different scenarios.

## 3 Projective Transformation

In a pinhole camera model, a point in 3D space is projected onto an imaging surface which is called an image plane (Kaehler and Bradski, 2016). All rays (or points) of light pass through a single point which is called a camera center. The size of the object on the image plane can be calculated from a similar triangle. Assuming the camera is calibrated or there are no distortions (radial and barrel distortion), a point (X,Y,Z) in the physical world is projected onto the image plane at (x,y) location with following equations:

x=fX/Z, (1)

y=fY/Z, (2)

where *f* is the focal length.

The relationship that maps a set of points from one image plane to a set of points to another image plane is called projective transformation. Planar homography is the projective mapping from one plane to another. According to Hartley and Zisserman (Hartley and Zisserman, 2003), projective transformation is defined as “A planar projective transformation is a linear transformation on homogeneous 3-vectors represented by a non-singular 3-by-3 matrix.”

$$\left(\begin{array}{c} x_{1}^{\prime }\\ x_{2}^{\prime }\\ x_{3}^{\prime } \end{array}\right)=\left[\begin{array}{ccc} h_{11} & h_{12} & h_{13}\\ h_{21} & h_{22} & h_{23}\\ h_{31} & h_{32} & h_{33} \end{array}\right]\left(\begin{array}{c} x_{1}\\ x_{2}\\ x_{3} \end{array}\right).$$ (3)

**FIGURE 1:**
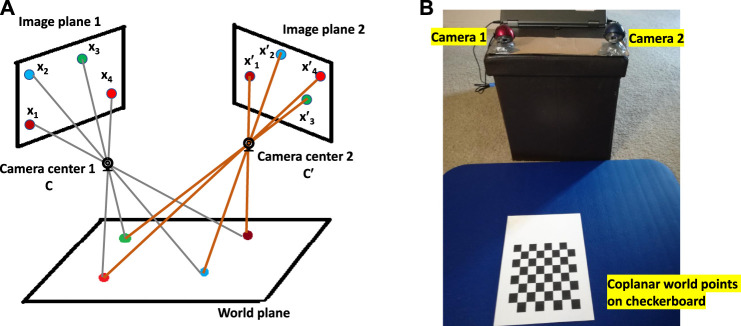
**(A)** For multiple cameras, image planes are related by projective transformation when all world points are coplanar. **(B)** Actual setup of two cameras with coplanar checkerboard points.

Or in short, $x^{\prime }=H.x$, where *H* is the homography matrix. This homography matrix relates the position of a point from a source image plane (image plane 1 in Figure 1A) to a destination image plane (image plane 2 in Figure 1A). $\left(x_{1}^{\prime }.x_{2}^{\prime },x_{3}^{\prime }\right)T$ are coordinates of a single point on two image planes. Also, *H* is a homogeneous matrix, which means only the ratio of the matrix elements is important. There are eight independent ratios in *H* ($h_{33}$ is a scaling factor), which means a projective transformation has eight degrees of freedom (Hartley and Zisserman, 2003).

Let us consider a pair of inhomogeneous matching points (x,y) and $\left(x^{\prime },y^{\prime }\right)$ on image planes 1 and 2, respectively. We are considering inhomogeneous coordinates because they can be measured directly from the image plane (coordinates of points in pixel). From Eq. 7.3:

$$x^{\prime }=\frac{x_{1}^{\prime }}{x_{3}^{\prime }}=\frac{h_{11}x+h_{12}y+h_{13}}{h_{31}x+h_{32}y+h_{33}},$$ (4)

$$y^{\prime }=\frac{x_{2}^{\prime }}{x_{3}^{\prime }}=\frac{h_{21}x+h_{22}y+h_{23}}{h_{31}x+h_{32}y+h_{33}}.$$ (5)

After rearranging:

$$x^{\prime }\left(h_{31}x+h_{32}y+h_{33}\right)=h_{11}x+h_{12}y+h_{13},$$ (6)

$$y^{\prime }\left(h_{31}x+h_{32}y+h_{33}\right)=h_{21}x+h_{22}y+h_{23}.$$ (7)

Four points on each image plane will create eight linear equations, and these four points are sufficient to solve for *H* between two image planes. If only four points are used, then the only condition is no three points can be colinear (Hartley and Zisserman, 2003). If more than four points are used, then no (n−1) points can be colinear, where *n* is the total number of points used. After the *H* matrix is calculated, it is then applied to the whole image plane 1 to convert it to image plane 2.

Important remarks:

1. Camera intrinsic parameters or pose are not needed to calculate *H.*
2. If only four points are used to calculate *H*, then outliers can create the incorrect *H* matrix.
3. Including more points and using a robust method to minimize a reprojection error will help to calculate the correct *H* matrix.

### 3.1 Homography Matrix Calculation

**FIGURE 2:**
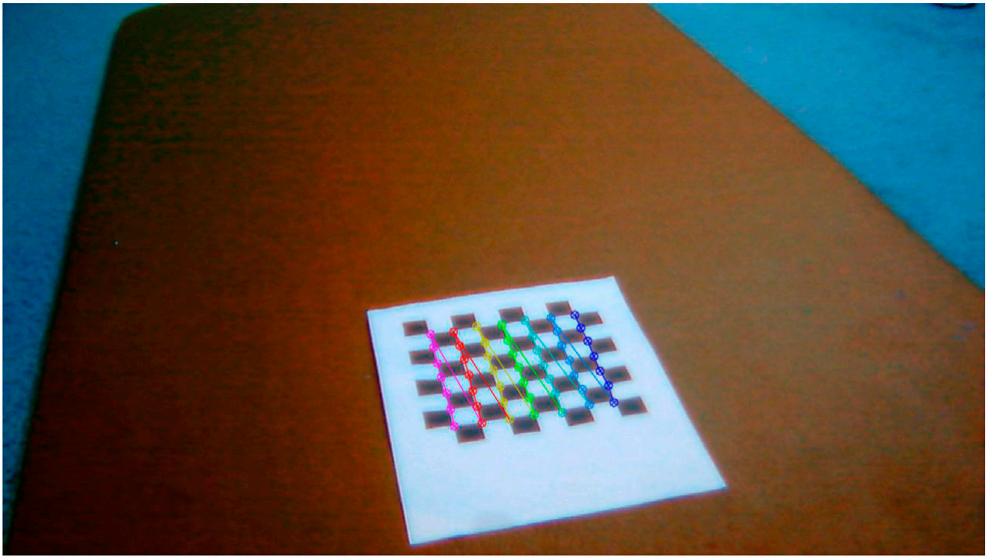
Checkerboard corner points for the left camera (camera 2).

**FIGURE 3:**
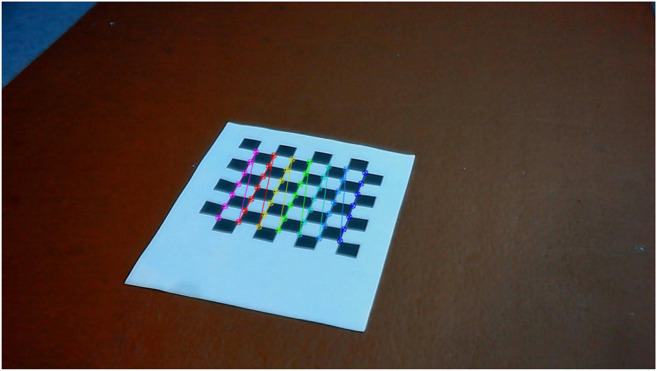
Checkerboard corner points for the right camera (camera 1).

We have established that no camera or pose parameters are needed to calculate the homography matrix, *H*. As a result, the following steps are followed to calculate *H*:

1. Place a printed checkerboard pattern in front of the cameras (Figure 1B).
2. Measure the pixel locations of checkerboard corners for each camera (Figure 2 and Figure 3).
3. Calculate the reprojection error which is the sum of squared Euclidean distances between *H* times the camera 2 checkerboard corner points and camera 1 checkerboard corner points.
4. Use an optimization algorithm to minimize the reprojection error for a specific *H* matrix.

We have tested four different optimization algorithms for *H* matrix calculation. The goal is to find a *H* matrix that minimizes the reprojection error. Least mean square minimizes the mean squared distance between checkerboard’s corner points of the two image planes. RANSAC is an iterative method, and it tries to find the correct corner points and eliminates the outlier corner points. PROSAC is a weighted RANSAC method, which is faster in case of many outliers. Least median square minimizes the squared median distances and more robust than least mean square when outlier is present. Then we calculated the projection error between the actual checkerboard corner locations and projected checkerboard corner locations. The error is calculated in terms of how much each corner point deviates from the actual corner location in terms of pixels. All results are presented in Table 1. Once the *H* matrix is calculated, that will remain the same as long as camera positions are constant. As Table 1 shows, both least mean square and least median square resulted in minimum reprojection errors. The *H* matrix calculated is

$$H=\left[\begin{array}{ccc} 9.29968576e-01 & -8.34785721e-01 & 3.28368011e+02\\ 3.21580417e-01 & 9.89425377e-01 & -3.15245367e+02\\ -7.93435882e-05 & 5.73575359e-05 & 1.0 \end{array}\right].$$ (8)

**TABLE 1:** Optimization methods and the reprojection error.

Method	Reprojection error (pixel/point)
Least mean square	1.621
RANSAC (Fischler and Bolles, 1981)	1.715
Least median square (Inui et al., 2003)	1.621
PROSAC (Bazargani et al., 2018)	1.961

**FIGURE 4:**
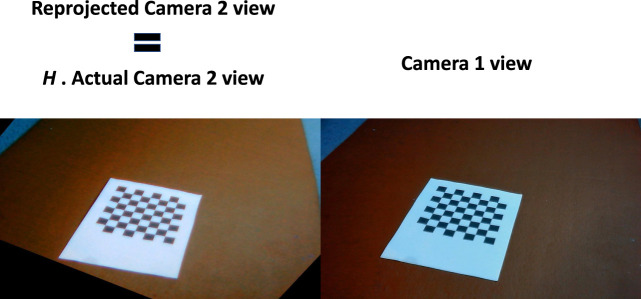
Reprojection of camera 2 image to camera 1 image using the homography matrix.

Figure 4 shows how the *H* matrix is used to reproject camera 2 view into camera 1 view. The left image of Figure 4 is the actual left camera view (Figure 2) multiplied by *H* which projects the left camera view into the right camera view.

## 4 CNN-Based Weed Detection

For this study, we considered a VGG16-based (Simonyan and Zisserman, 2014) model architecture with transfer learning. This model is retrained using a small weed dataset, and final layers are rearranged to classify three different weeds. Transfer learning retrains the final layers of the VGG16 model to classify new objects from a new dataset by using the large amount of features already learned from the ImageNet database. Previous research (Karpathy et al., 2014; Mohanty et al., 2016) has shown that transfer learning has much lower computational requirements than learning from scratch and can be applied to various types of classification. All the training simulations are run on an Intel core-i7, 8gb ram, Nvidia GTX 1060 6gb workstation. Models are built with Keras library on TensorFlow backend in *Python* 3.5.

Images of common cocklebur, redroot pigweed, and giant ragweed are captured to generate a weed dataset. These three types of commonly found corn weeds were grown in the IUPUI Greenhouse in order to collect images. Also, images were captured from actual corn fields. Maximum input image size used in this study is 150-by-150 pixels (input dimension of the CNN network).

### 4.1 Transfer Learning With VGG16

VGG16 is a 16-layer CNN which introduced the idea of multiple small kernel filters. Trained on the ImageNet dataset from 2012, it was able to classify 1,000 classes. The top-5 error rate of VGG16 was 7.4% (Simonyan and Zisserman, 2014). The model parameters implemented in this study include learning rate (1e-05), optimizer (Adam), and loss (sparse categorical cross-entropy).

Now, we fit a detector on top of the classifier to get the BB. We should mention that we are just fitting a detector, and we are not training the detector for better performance. First, an image pyramid is created to deal with different scale factors. The reason is to detect objects at different scales. As the image pyramid grows bigger, it will help to detect bigger objects. Then we run a sliding window at each scale of the pyramid. The size of the sliding window should depend on the size of the object to be detected. At each position of the sliding window, we are passing it through the CNN classifier and saving the class label with % accuracy. Basically, we are creating a lot of bounding boxes with class label accuracy and saving their location. Then we pass all the BBs through an algorithm called non-maximum suppression (NMS). NMS takes a BB, and then calculates IOU with all the other BBs. If the IOU value is over some predetermined threshold, then that BB is discarded, else that BB is kept. Basically, NMS tries to figure out which one is the unique BB. These steps are standard and close to the steps followed in SSD (Liu et al., 2016) but without the training.

From classification report in Table 2, we can say that Cocklebur shows near-perfect recall. Pigweed is the hardest one to classify as the CNN model shows only 0.89 recall. Inference time is an important parameter for real-time classification from the video feed. This inference time (without GPU acceleration) seems adequate (about four frames per second (FPS)) for our test case. With GPU acceleration (Nvidia GTX 1060 Ti), this model performs at around 30 FPS.

**TABLE 2:** Classification report of the trained CNN model. Inference time tested on a Core-i5, 8 gb ram machine.

	Cocklebur	Pigweed	Ragweed
Precision	0.94	0.94	0.96
Recall	1.0	0.89	0.94
$F_{1}$ score	0.96	0.94	0.95
Training accuracy	0.99		
Validation accuracy	0.97		
Testing accuracy	0.94		
Inference time	0.266°s		

### 4.2 IOU Overlap Calculation

Now that we have the BB from the detector, we want to calculate the IOU value for left and right camera BBs. We want to see how the IOU value changes before and after reprojection when weed is detected correctly and the BB is created. We also want to see how the IOU value changes when weed is detected at different distances from the camera.

**FIGURE 5:**
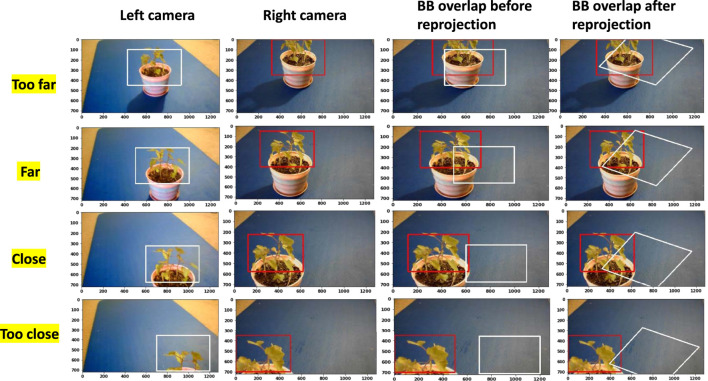
How BB overlap changes when weed is placed at different distances from the camera. “too far,” “far,” “close,” and “too close” represent the distance of the weed from the camera. “After reprojection” shows the BB overlap when the left camera image is reprojected using *H*. BB size, width: 500 pixels; height: 350 pixels.

Figure 5 shows how BBs from both cameras overlap each other at different distances. “Too far” is about 36 inches away from the camera, and “too close” is about 8 inches away from the camera. BB sizes are fixed for both the cameras. We also assume perfect detection. As the weed moves closer to the camera, we can see that the BB overlap is decreasing, and for the “Too close” position, without reprojection, the BB overlap is zero. With reprojection (the left camera image is reprojected using *H*), the BB overlap still decreases as weed moves closer to the camera. Homography is 2D transformation from one camera image plane to another camera image plane. But the object (weed) we are detecting is actually 3D. As a result, as we move closer to camera, BB overlap decreases because projection of the 3D object onto a 2D plane deviates more. If the objects we are detecting were 2D then after reprojection, BB overlap would remained more or less the same.

**FIGURE 6:**
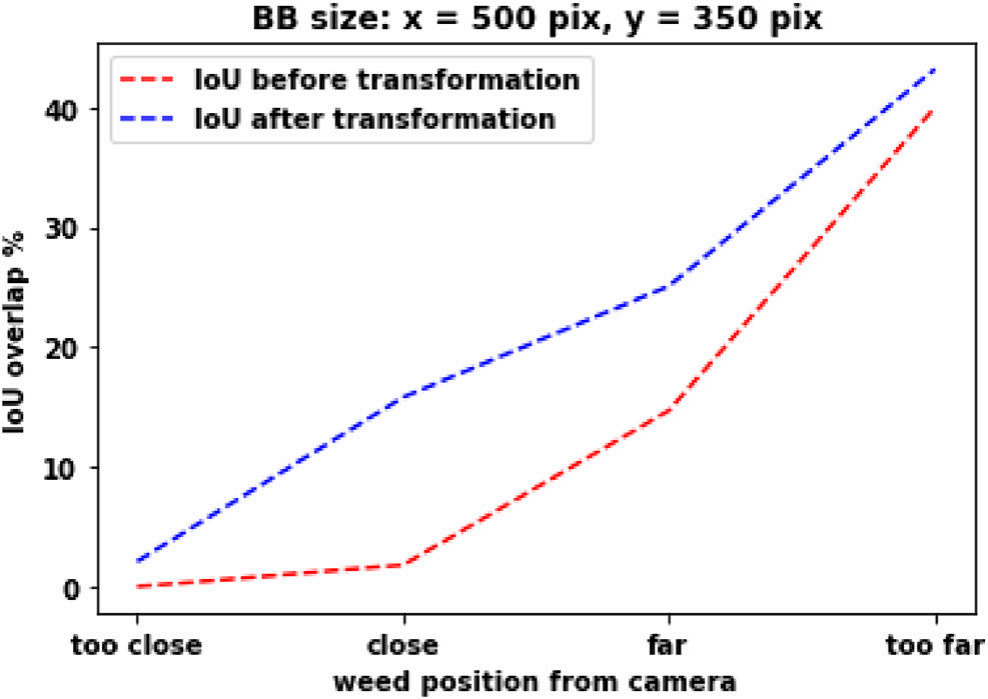
IOU overlap value with respect to distance from the camera. **(A)** BB size, width: 500 pixels; height: 350 pixels. **(B)** BB size, width: 450 pixels; height: 300 pixels.

Figure 6 shows how the IOU value changes when weed distance (position of BB) changes. As an example, for “close” position and for perfect detection, the IOU overlap between two cameras should be 15%. The IOU overlap after reprojection is always bigger than that before reprojection. As we move farther, the difference between two different systems reduces. In the actual scenario, a CNN detector will create a BB based on the size or shape of the object, and they will be different based on the scenario. But for this study, we have kept the BB size constant (BB width: 500 pixels, height: 350 pixels). But in future, different BB sizes could be considered, and the IOU value should be calculated for a range of BB sizes.

## 5 Fuzzy Logic–Based Fusion

**FIGURE 7:**
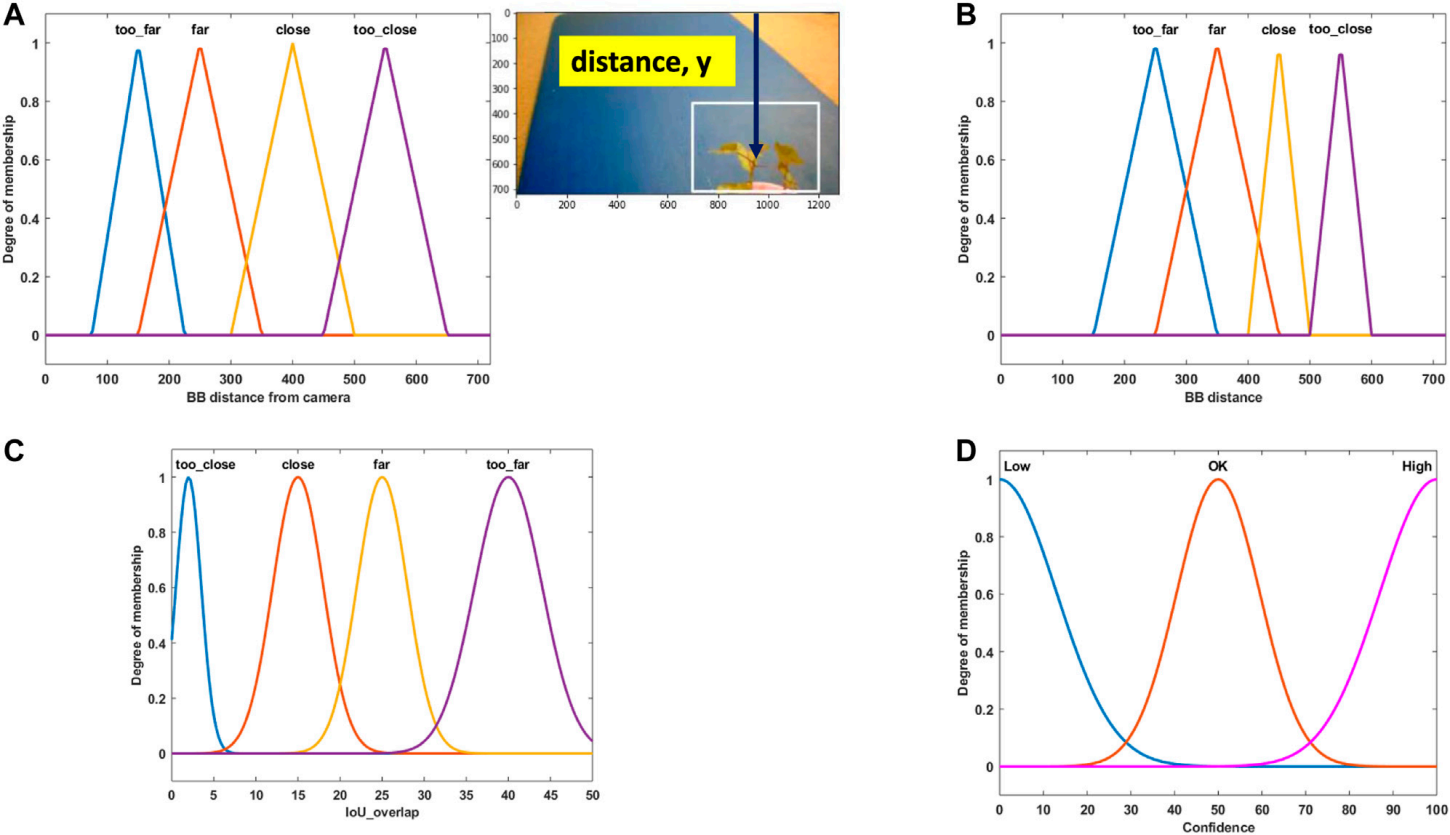
**(A)** Fuzzy set for the weed bounding box distance in camera view for the right camera [Input]. **(B)** Fuzzy set for the weed bounding box distance in camera view for the left camera [Input]. **(C)** Fuzzy set for bounding box overlap between the left and right cameras [Input]. **(D)** Fuzzy set for fusion confidence [Output].

Fuzzy logic was invented by Lotfi Zadeh (Zadeh, 1965) by combining crisp logic and set theory. In reality, many concepts are better defined by human words than by mathematics. Zadeh tried to capture that link between human language and mathematics. Zadeh used fuzzy sets to capture that relationship. If there is uncertainty about membership data regarding those data belonging to a particular set, fuzzy sets are used to define those data to a partial set: as an example, if fuzzy sets are used to define the bounding box position of weed that can be defined as “too close,” “close,” “far,” or “too far”; and for confidence of fused bounding box as “low,” “ok,” and “high.” The membership degree quantifies the level of how those data belong to that set. As an example, in the case of bounding box distance from the camera center:

$$m_{dis\tan ce}\left(y\right)\in \left[0,\;1\right],$$ (9)

where $m_{distance}\left(y\right)$ is the degree of membership *y* has in fuzzy set of “distance in camera view” and *y* is the vertical distance from top of the camera view in pixel value. Figure 7A shows this.

Membership function expresses various degrees of strength between the elements in fuzzy set. If likelihood is higher that an element belongs to a certain set, then the membership strength is also higher. Membership strength of zero means that the element does not belong to that set, and membership strength of one means that the element definitely belongs to that set. In this study, fuzzy sets are used to define the distance of bounding box, IOU overlap, and confidence in the bounding box position.

The membership function design is based on a combination of our personal experience and the knowledge we gained from the camera setup testing. In Figures 7A,B, the membership function for the right and left camera bounding box position is presented, respectively. Weed is placed at four different distances in front of the camera, as shown in Figure 5. Fuzzy names are selected based on their position (“too far,” “close,” *etc*.). Triangular membership functions are selected to represent their strength. As an example, in Figure 7A, for “too far” position, the BB distance has a maximum degree of membership (one) at position 150 (pixel), which means when weed is placed at “too far” position and the best BB is generated by the CNN detector, the BB y-position center (vertical distance) is found at 150 pixels from the top position of the camera view. In Figure 7C, the membership function for IOU overlap is presented. It is derived from Figure 6. As an example, for the “close” position, the IOU overlap value from Figure 6 is 15%, which means that when weed is placed at the “close” position and BB is created from the CNN detector, for perfect detection (best possible fit of BB over the weed), the IOU overlap value will be 15%. The overlap value will be different if it is not a perfect detection at this position. The Gaussian membership function is used to represent this. Figure 7D shows the confidence membership function. If the BB distance and IOU overlap value perfectly match, then they will have “high” confidence and based on their deviation from perfect condition and the fuzzy rule set, they can be “ok” or “low.”

### 5.1 Fuzzy Steps and Rule Set

**FIGURE 8:**
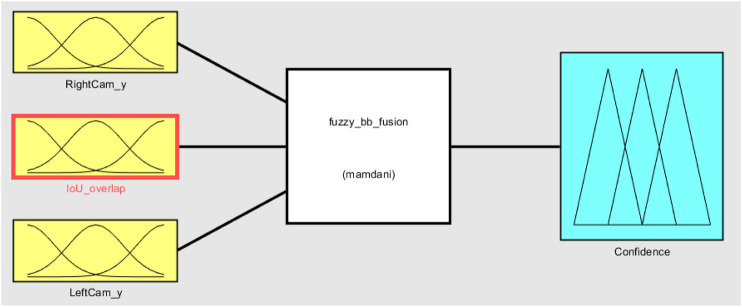
Basic configuration of the fuzzy system.

The overall fuzzy system with inputs, rule evaluations, and defuzzification (output) is presented in Figure 8. Following steps are followed for the application of fuzzy analysis:

1. Identify inputs with their ranges and name them: In this study, the subsets for weed distance and IOU overlap are too close, close, far, and too far.
2. Identify output with their ranges and name them: In this study, the subsets for confidence are low, ok, and high.
3. Create a degree of the fuzzy membership function for inputs and outputs: Figure 7 shows this.
4. Construct the rule base for the system based on expert judgment: The rules creates a linguistic relationship between the input variables and the output. In a fuzzy “IF..THEN” rule, the IF part is the premise and the THEN part is the output based on premise. The rules can be combined with logical “OR” or logical “AND.” Here, the three input variables are the following: RightCam BB (right camera BB distance), LeftCam BB (left camera BB distance, and IOU overlap, and the output variable is Confidence. The generic conditional statement used in this study is as follows:

$$\begin{array}{cc} R_{n}:IF\;Camera_{i}\;BB\;is\;A\left(n\right)\;and\;Camera_{j}\;BB\;is\;B\left(n\right) & \\ and\;IOU\;overlap\;is\;C\left(n\right) & \\ THEN\;Confidence\;is\;D\left(n\right). & \end{array}$$ (10)

where i≠j; i,j are the number of cameras; A(n), B(n), and C(n) are too close, close, far, and too far; and D(n) is low, ok, and high. Twenty fuzzy rules were designed to optimize for the relationship between output and inputs. All rules are not valid for this problem. Some rules might not get triggered at all based on the BB position and IOU overlap value. All rules are given equal weight of one. The final output, that is, Confidence, is the union of the output fuzzy subsets for the activated rules. In this research, the Mamdani (Mamdani, 1974) inference is used. All the rules are presented in Table 3.

5. Defuzzification: A centroid defuzzification method is used in this research (Ross et al., 2004).

**TABLE 3:** Fuzzy rules.

	Rules description	
$R_{1}$	IF (RightCam BB is too far) and (IOU overlap is too far) and (LeftCam BB is too far) THEN	(Confidence is High)
$R_{2}$	IF (RightCam BB is far) and (IOU overlap is far) and (LeftCam BB is far) THEN	(Confidence is High)
$R_{3}$	IF (RightCam BB is close) and (IOU overlap is close) and (LeftCam BB is close) THEN	(Confidence is High)
$R_{4}$	IF (RightCam BB is too close) and (IOU overlap is too close) and (LeftCam BB is too close) THEN	(Confidence is High)
$R_{5}$	IF (RightCam BB is too far) and (LeftCam BB is too close) THEN	(Confidence is Low)
$R_{6}$	IF (RightCam BB is too far) and (LeftCam BB is close) THEN	(Confidence is Low)
$R_{7}$	IF (RightCam BB is too far) and (LeftCam BB is far) THEN	(Confidence is Low)
$R_{8}$	IF (RightCam BB is far) and (LeftCam BB is too far) THEN	(Confidence is Low)
$R_{9}$	IF (RightCam BB is far) and (LeftCam BB is close) THEN	(Confidence is Low)
$R_{10}$	IF (RightCam BB is far) and (LeftCam BB is too close) THEN	(Confidence is Low)
$R_{11}$	IF (RightCam BB is close) and (LeftCam BB is too far) THEN	(Confidence is Low)
$R_{12}$	IF (RightCam BB is close) and (LeftCam BB is far) THEN	(Confidence is Low)
$R_{13}$	IF (RightCam BB is close) and (LeftCam BB is too close) THEN	(Confidence is Low)
$R_{14}$	IF (RightCam BB is too close) and (LeftCam BB is close) THEN	(Confidence is Low)
$R_{15}$	IF (RightCam BB is too close) and (LeftCam BB is far) THEN	(Confidence is Low)
$R_{16}$	IF (RightCam BB is too close) and (LeftCam BB is too far) THEN	(Confidence is Low)
$R_{17}$	IF (RightCam BB is too far) and (IOU overlap is Not too far) and (LeftCam BB is too far) THEN	(Confidence is OK)
$R_{18}$	IF (RightCam BB is far) and (IOU overlap is Not far) and (LeftCam BB is far) THEN	(Confidence is OK)
$R_{19}$	IF (RightCam BB is close) and (IOU overlap is Not close) and (LeftCam BB is close) THEN	(Confidence is OK)
$R_{20}$	IF (RightCam BB is too close) and (IOU overlap is Not too close) and (LeftCam BB is too close) THEN	(Confidence is OK)

## 6 Results

Three scenarios are created and tested to check the performance of the fuzzy system. In all the following scenarios, we are assuming that the CNN detector is detecting the weed with 100% accuracy inside the BB, the whole weed plant is visible from both cameras, and there is no occlusion.

### 6.1 Scenario 1: High Confidence

**FIGURE 9:**
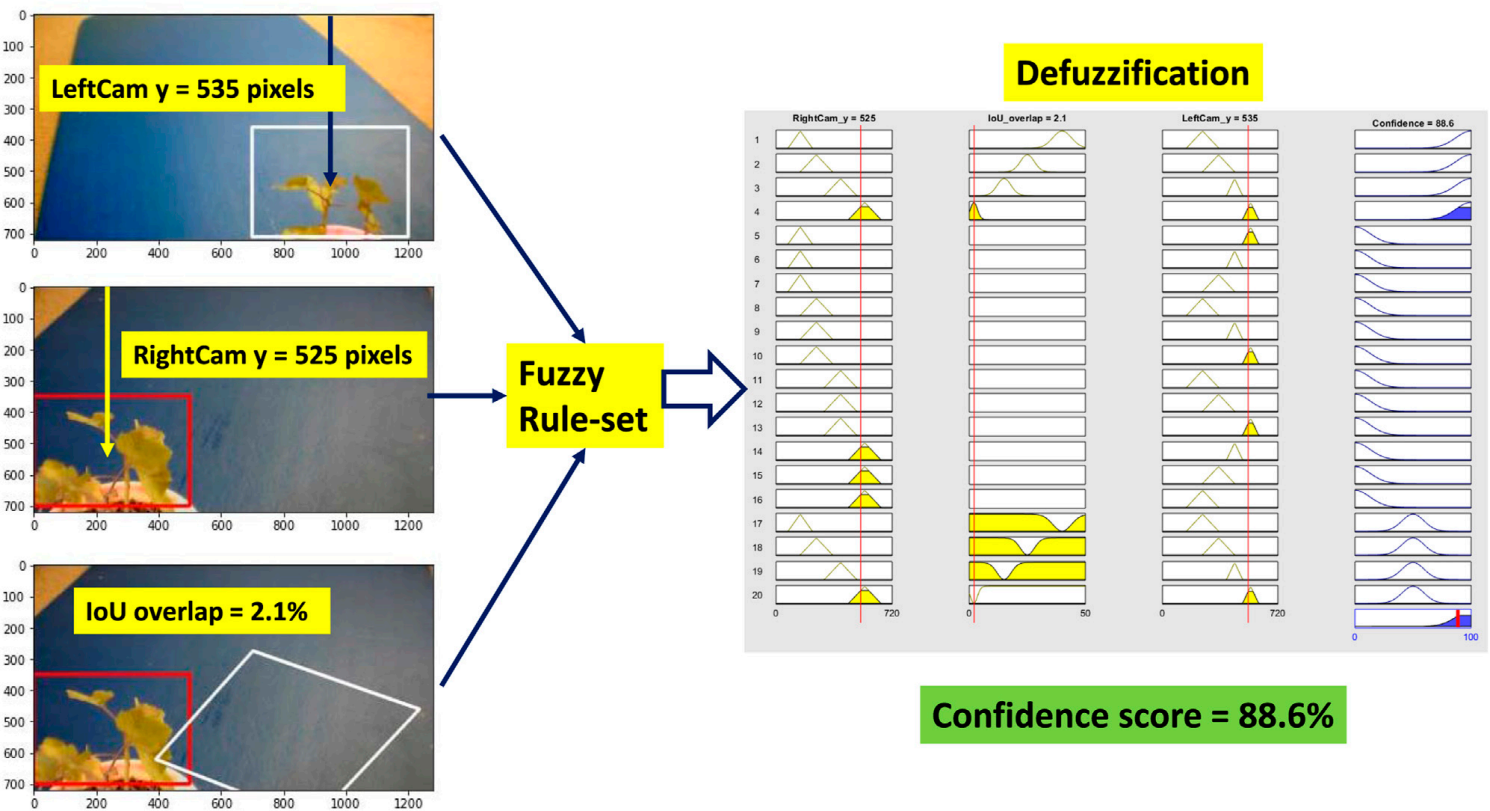
Fuzzy confidence score measurement (high confidence). Left-hand side images show the position of detected BB and IoU overlap. Then the position value of the BB and IoU overlap goes into the fuzzy rules defined in Table 3. After the defuzzification step, we receive the confidence score of the detection.

Weed at position “too close” is chosen to test the performance of the system. Figure 9 shows all the steps of the fuzzy confidence score measurement system. For “high confidence” situation, the CNN detector detects the weed correctly. The BB covers the whole weed for both cameras, and the weed is usually at the center of the BB. As three inputs, we measure the vertical center distance of the BB for both the cameras and the IOU overlap score. All three inputs goes into the fuzzy rule set. For a specific position of the weed in the 3D world, if the weed is detected by both cameras correctly, then they should have a specific IOU overlap value. As a result, all these inputs trigger the fuzzy rule four ($R_{4}$ in Table 3). This follows one of the rules for high confidence. After defuzzification, we receive a confidence score of 88.6%. The fuzzy system gives a high confidence score because the weed is detected correctly by both the cameras. One important thing is because we are using the Gaussian membership function and centroid defuzzification, high confidence score will always provide a value between 70 and 100% but not exactly 100% even if the detection meets all the criteria (rules) for perfect detection.

### 6.2 Scenario 2: OK Confidence

**FIGURE 10:**
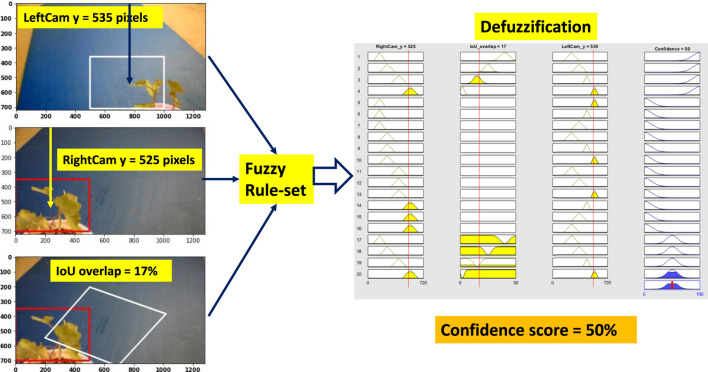
Fuzzy confidence score measurement (ok confidence).

Weed at position “too close” is chosen to test the performance of the system. Figure 10 shows all the steps of the fuzzy confidence score measurement system for “ok confidence” situation. Here, the right camera detects the weed correctly, but the left camera detection is partially correct and detecting the weed at a slightly left position than the actual position. However, the BB center vertical distance for both right and left cameras is correct and comparable to “high confidence” situation. But because the left camera is detecting at a partially correct position, this deviates the IOU overlap value and triggers rule 20 ($R_{20}$ in Table 3). As a result, the fuzzy system gives it a confidence score of 50% which is a reasonable value given the partial detection.

### 6.3 Scenario 3: Low Confidence

**FIGURE 11:**
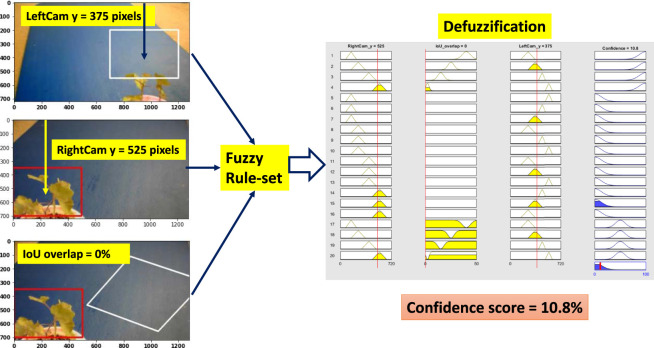
Fuzzy confidence score measurement (low confidence).

Weed at position “too close” is chosen to test the performance of the system. Figure 11 shows all the steps of the fuzzy confidence score measurement system for “low confidence” situation. Here, the right camera detects the weed correctly, but the left camera detection is less than partially correct and detecting the weed at a higher position than the actual position. As a result, the BB center vertical distance is not comparable with the “high confidence” situation. This triggers rule 15 ($R_{15}$ in Table 3). As a result, the fuzzy system gives it a confidence score of 10.8% which is a reasonable value given the less than partial detection.

One limitation of this fuzzy confidence measurement system is this inherently assumes one of the camera detection is correct. But if both of them are incorrect, then this system may produce a less than ideal confidence score. There are two ways to tackle this limitation. First, for video input, we can incorporate a tracking algorithm for each BB. If a BB position deviates more than a threshold value or more than the previous position, and does not follow a trend, then we can either discard that frame or discard that BB from the fuzzy system to produce better results. Second, we can incorporate more than two cameras into the system. Then we can calculate the evidence distance (in this case BB position) for each camera and then discard or give lower weight to the BB which deviates from usual norm. If we incorporate these into the fuzzy system, then the system becomes more complex and loses the inherent advantage of a fuzzy system which is easy to interpret. If more than two cameras are used, then it is recommended to use uneven number of cameras ((2n+1) numbers of cameras, where n=1,2,3,…). As an example, if five cameras are used, then the *H* matrix should be calculated to reproject the other four camera planes onto the center camera plane. IOU overlap values should be recalibrated based on new test cases. Basic configuration (Figure 8) will change. The inputs will be “LeftCam-1-y,” “LeftCam-2-y,” “CenterCam-y,” “RightCam-1-y,” “RightCam-2-y,” and “IOU-overlap.” But the number of fuzzy rules will increase with increasing number of cameras.

## 7 Conclusion

In this research, we developed and used a fuzzy logic–based fusion algorithm to calculate the confidence score of the BB position and IOU overlap obtained from a multi-camera–based CNN object detector. First, a CNN-based object detector was used to detect weed at multiple positions in front of the multi-camera setup. Then a projective transformation was used to project one camera’s image onto another camera’s image plane. Then we calculated the IOU overlap value at each of the different positions of the weed for perfect detection. When detecting an object using multiple cameras, the position of the BB on the camera image plane may appear in different places based on the detection accuracy and the position of the cameras. But in 3D space, the object is at the exact same position for all cameras. As a result, a relationship can be established between the IOU overlap value of the BBs and the position of the BBs on a camera image plane. If the BB position or the IOU overlap value deviates from the ideal condition, then that would indicate a less than perfect detection. We generated a fuzzy rule set using this relationship between the BB position of each camera and IOU overlap value. The proposed fuzzy system was tested for three different scenarios, which are ideal detection (high confidence), less than ideal detection (ok confidence), and wrong detection (low confidence). The confidence score of the proposed fuzzy system for three different scenarios proved the hypothesis regarding a relationship between IOU overlap and BB position and offered a more robust overall multi-camera–based object detection system.

## Data Availability

The raw data supporting the conclusions of this article will be made available by the authors, without undue reservation.
